# Leading Change in Health Education: Assessing Organisational Readiness to Implement Simulation in Multidisciplinary Programmes

**DOI:** 10.1155/jonm/9136330

**Published:** 2026-07-27

**Authors:** James Naismith, Wendy Cross, Kerry Hood, Loretta Garvey

**Affiliations:** ^1^ Institute of Health and Wellbeing, Federation University, Berwick, Victoria, Australia, federation.edu.au

## Abstract

**Background:**

Simulation‐based education (SBE) is recognised as a highly effective approach for preparing healthcare students for clinical practice. However, its consistent integration and long‐term sustainability remain challenging, often due to insufficient resources, limited faculty capability and the absence of robust educational frameworks. Organisational readiness, which is an important yet under‐examined determinant of SBE success, plays a critical role in overcoming these barriers, particularly in multidisciplinary and multi‐campus contexts.

**Objective:**

This study aimed to evaluate an organisation’s readiness to implement SBE programmes across diverse health disciplines by applying the Simulation Culture Organisational Readiness Survey (SCORS).

**Methods:**

The SCORS tool, comprising four subscales and 38 Likert‐scale items, measures readiness for change, availability of resources and capacity for sustainable development. A cross‐sectional survey was conducted via Qualtrics XM, supplemented with demographic data to account for participants’ disciplinary backgrounds.

**Results:**

A total of 43 staff completed the survey, with the largest representation from Nursing (46%) and Physiotherapy (14%). The highest mean score was observed in Section A—Need and Support for Change (3.04), indicating a strong perceived value for SBE. Conversely, Section C—Time, Personnel and Resource Readiness—recorded the lowest mean score (2.31), signalling significant operational challenges. The overall readiness score of 103.86 placed the organisation as ‘somewhat ready’ for SBE integration, with administrators identified as key influencers in the adoption process.

**Implications for Nursing Management:**

Findings highlight the need for a centralised, strategically governed SBE framework supported by sufficient resourcing, embedded leadership and faculty development. Clear reporting structures, coordinated resource allocation and a service‐oriented operational model are recommended to ensure interdisciplinary collaboration and sustainability across campuses. These findings have direct implications for nursing management, particularly for nurse education leaders, nursing simulation programme managers and academic nursing managers responsible for aligning simulation governance, faculty workload allocation, capability development and curriculum integration within nursing and wider health programmes.

**Conclusion:**

Achieving sustainable SBE integration requires a whole‐of‐organisation approach that aligns governance, pedagogy and operational capacity, ensuring that simulation becomes a core, enduring component of health professional education.

## 1. Introduction

Simulation‐based education (SBE) is an effective and widely accepted approach for preparing undergraduate healthcare students for professional practice [1, 2]. While the effectiveness of SBE is well established, the success and viability of SBE programmes vary widely [3]. There are many reasons for this variability and by identifying the challenges and building upon the enablers, SBE programmes have a greater chance of being embedded into curriculum and sustained in pre‐registration curricula [3, 4]. This requires attention to both the initial implementation of SBE, including its deliberate alignment with curriculum, learning outcomes, clinical competencies, assessment requirements and learner progression, and its ongoing sustainability as an integrated, adaptable and institutionally supported component of education. The successful implementation and sustained integration of SBE in healthcare programmes depend on adequate resource allocation and comprehensive faculty training and development [4, 5]. However, without the implementation of robust simulation design frameworks grounded in evidence‐based pedagogical principles, SBE initiatives may lack effectiveness and fail to sufficiently prepare students for clinical practice. In addition, contemporary nursing education literature further emphasises the importance of innovative, student‐centred teaching models, such as flipped classroom approaches, which have been shown to enhance engagement, critical thinking and learning outcomes [6]. This highlights the necessity of embedding SBE into curriculum with innovative and well‐structured frameworks to ensuring institutional support. The use of pedagogically sound simulation design frameworks appears to be one of the most critical components of successful SBE implementation [5].

Robust organisational support for both management and faculty is critical in developing a compelling business case to optimise the procurement and allocation of resources, encompassing both physical and human assets [4]. Such support also underpins the establishment of an academic and professional simulation faculty endowed with specialised skills. These skills are distinctive and indispensable to the effective delivery of SBE programmes [7]. The ongoing success and sustainability of SBE programmes are dependent upon an organisational commitment to support a structured programme across all levels of the organisation, and without these support frameworks in place for management, compromises the survival of SBE programmes [4, 7].

To effectively understand organisational and management needs, there is a requirement to gather baseline data to understand the staff’s requirements, perceptions and identified gaps [8]. Assessing organisational readiness is crucial when implementing new initiatives, policies and programmes into an organisation, as it helps gain an insight into psychological and structural perceptions, identifies factors contributing to readiness and provides an opportunity for management to address the readiness gap [9, 10].

There is limited research on the influence of demographics and cultural factors on organisational readiness for formal SBE programmes. Therefore, readiness needs to be considered beyond individual disciplines and examined within the broader organisational landscape, particularly where SBE is implemented across multiple health professions and campuses [11–13]. Cook et al. [12, 14] demonstrate the scale and complexity of the simulation evidence base, including the challenges of synthesising findings across diverse health professions, settings, interventions and outcomes [14]. Similarly, Cook et al. [11] argue that the value of simulation should be considered in relation to educational outcomes, resource investment and broader organisational impact. Accordingly, this study does not attempt to examine all possible uses of simulation across healthcare education. Instead, it focuses on selected pre‐registration health disciplines within one multi‐campus university, allowing organisational readiness, governance, resourcing, faculty capability and sustainability to be examined at an institutional level.

However, further research is required to explore the skills, training and ongoing professional development required for their effective implementation and ongoing sustainability [15].

### 1.1. Aim of the Study

The aim of this study was to evaluate an organisation’s readiness to implement SBE programmes through the application of an organisational readiness assessment tool, while accounting for local demographic factors, in order to identify the organisational barriers and facilitators to implementation.

## 2. Methods

The study used a cross‐sectional survey design and used the Simulation Culture Organisational Readiness Survey (SCORS) tool [16] together with a demographic question set, identifying the health discipline of participants.

The SCORS tool is a survey designed by Leighton et al. [16] and was developed to assist organisations in evaluating institutional and programme readiness for simulation integration [16]. It was designed to assist organisational management to better understand the components of organisational readiness prior to any decisions about investment into simulation programmes, with the goal of increasing effective and efficient integration of simulation into the academic curriculum [16, 17].

The SCORS combines four tools: the Organisational Culture and Readiness for System‐Wide Integration of Evidence‐based Practice (EBP) Survey (OCRSIEP) [18], the Team Strategies and Tools to Enhance Performance and Patient Safety (STEPPS) Organisational Readiness Assessment Checklist [19], Organisational Elements that Shape Simulation in Nursing Model (OESSN) [20] and the Capacity Building for Simulation Sustainability Model (CBSS) [17]. By combining these four instruments, the SCORS tool is designed to sort the content areas into themes that pertain to the readiness of the organisation allowing for easier exploration [21].

The tool consists of 38 questions, each rated on a five‐point Likert scale from 1 to 5 (1: none at all to 5: very much), and if a mean falls below 3, this is indicative of an opportunity for intervention. The scores are added together to provide a cumulative total, which indicates the level of readiness for the organisation, with 0–36 being not ready, 37–72 a little, 73–108 somewhat, 109–144 moderately and 145–180 very much (Table 1).

**TABLE 1:** SCORS interpretation score.

Interpretation	Overall SCORS	Defined need and support for change	Readiness for culture change	Time, personnel and resource readiness	Sustainable education development to embed culture
None	0–36	0–9	0–11	0–12	0–4
A little	37–72	10–18	12–22	13–24	5–8
Somewhat	73–108	19–27	23–33	25–36	9–12
Moderately	109–144	28–36	34–44	37–48	13–16
Very much	145–180	37–45	45–55	49–60	17–20

It is divided into four subsections that evaluate various aspects of organisational readiness for cultural change. The subsections include defined need and support for change, readiness for culture change, time, personnel and resource readiness and sustainable education development to embed culture. In addition to the subsections, there is a summary section that includes two questions. This section provides an overall impression and a total score, which categorises the organisation’s readiness to embed an SBE programme. The first 36 items are used to calculate an overall score, which can range from 36 to 180. The SCORS was developed and psychometrically evaluated by Leighton et al. [16], with published psychometric evidence supporting its use in assessing organisational readiness for simulation integration [16, 22]. In the original study, the complete SCORS demonstrated excellent internal consistency, with Cronbach’s alpha of *α* = 0.96, while subscale reliability ranged from *α* = 0.71 to *α* = 0.92. Subsequent studies have further supported the use of SCORS within nursing education contexts in terms of reliability with Cronbach’s alpha ranging from *α* = 0.89 to *α* = 0.97 [23, 24]. The present study did not seek to generate further validity evidence for the instrument. Reliability was assessed in the current sample, with the total SCORS scale demonstrating excellent reliability, *α* = 0.945, and subscale reliability ranging from *α* = 0.821 to *α* = 0.853. The tool uses a scoring matrix to identify areas of strengths and weaknesses that may impact, thus providing insight into the staff’s perception and the organisation’s readiness for implementing an SBE programme into practice.

In this research, the SCORS tool was used to collect data to explore the perceptions of staff about the readiness of the organisation to implement SBE within a university based in Victoria, Australia [16]. The survey was administered via Qualtrics XM online survey software [25], which enabled the survey to be accessed via a unique link, facilitate the collection of deidentified data and analysis of responses within the one secure platform. Qualtrics incorporates advanced tools to minimise invalid or automated entries. These include CAPTCHA challenges and automated systems that detect and block unusual behaviours—such as extremely fast completion times, repeated entries from a single IP address or erratic answer patterns. These protective features worked collectively to ensure the reliability of the dataset by filtering out bots, deceptive participants and individuals not aligned with the intended respondent group. Adopting a non‐experimental design approach allowed for scrutiny of current practices and perceptions of staff and was deemed the most effective approach as it recognises the subjective nature of the problem and different experiences of the participants [26].

### 2.1. Research Participants

The study was conducted at a university based in Victoria, Australia, which consists of both metropolitan and regional campuses, and the study sample included both academic and professional staff who are employed in a permanent capacity. Professional staff included technical specialists, administrative assistants and administrators. Administrators were defined as senior personnel responsible for the oversight of academic courses, processes and related activities, including the allocation of resources and contribution to the strategic direction of these functions. Only permanent staff were included as they were considered to have a greater understanding of the organisational context and the most organisational knowledge. Sessional staff were excluded as it was considered their understanding of the organisational context would be more limited and possibly less accurate: Staff from Nursing, Midwifery, Paramedicine, Occupational Therapy, Speech Pathology, Exercise Sports Science, Physiotherapy, Psychology and Public Health participated. The focus of this study was SBE in health disciplines; therefore, non‐health disciplines were excluded.

### 2.2. Ethical Considerations

The application to undertake this research project was deemed to be low risk and was approved by the University Human Research Ethics Committee (Reference number 2024/044 V2).

Participants in the research were provided with a plain language information sheet (PLIS), which provided precise, non‐technical information and clear common language explanations regarding the study. Consent was implied by the returning of the completed survey and was explicitly stated in PLIS document. Any information obtained in connection with this research project was deidentified, and all the data collected and stored were not identifiable. Electronic data were stored in a password‐protected file at a university‐secured server, and information was only accessed by members of the research team.

### 2.3. Data Collection

An invitation to participate in the research was emailed to the participants with accompanying documentation, and participants were asked to complete the surveys. This was distributed by the Academic Support services team to avoid any perception of coercion from the research team. No personal information was collected, and any potential identifying data were deidentified. Each survey was expected to take between 5 and 10 min to complete.

The survey was distributed to 116 permanent staff members across the healthcare disciplines and across multiple campuses. Forty‐four surveys were returned; one survey was incomplete and therefore not included in the analysis. This resulted in 43 completed surveys included in the analysis, with a response rate of 37%.

### 2.4. Data Analysis

Upon completion of the data collection, data were aggregated, exported and evaluated using the Statistical Package for Social Science (SPSS) [27].

The data were analysed using descriptive statistics, allowing for both tabulation of the data and statistical interpretation [28]. The results were separated into the four subscales of (a) defined need and support for change, (b) readiness for culture change, (c) time, personnel and resource readiness and (d) sustainability practices to embed culture. Determining factors were then identified from the analysis of the data and were discussed to identify barriers and enablers in the responses.

## 3. Results

Although the study was designed within a multidisciplinary and multi‐campus framework, subgroup sizes were small and variability between groups was minimal, limiting meaningful subgroup comparison. Accordingly, the data were analysed as a single cohort to support organisational‐level interpretation.

The data showed that there was a contribution of survey returned from all disciplines and a total of 43 staff members comprised the final dataset. Most responses were from Nursing (46% of total responses) and the second highest from Physiotherapy (14% of total responses), which was a representation of the population size. (See Table 2).

**TABLE 2:** Survey response rate by discipline.

Discipline	Response rate *n* (%)
Speech Pathology	3 (75)
Public Health	1 (25)
Physiotherapy	6 (60)
Exercise and Sports Science	2 (16)
Psychology	5 (20)
Occupational Therapy	2 (33)
Paramedicine	1 (50)
Midwifery	3 (50)
Nursing	20 (42)
Total	43 (37)

*Note:* Percentages represent the response rate within each discipline, calculated using the number of completed surveys returned from each discipline divided by the number of eligible staff invited from that discipline. Percentages do not represent each discipline’s proportion of the final sample.

The following results are presented by separating the data into the four subcategories within the SCORS tool. Each individual question has been scored from 1 (not at all) to 5 (Very Much), and a mean calculated for each question.

Section A of the SCORS tool addresses the defined need and support for change. Question 8, To what extent have the educators you work with articulated a need for SBE integration into the curriculum, returned the highest score (Question 8, Mean = 3.72) followed by question 1, To what extent are innovation, experiential learning and quality student experiences clearly described as central to the mission and philosophy of your institution (Question 1, mean = 3.65) (see Table 3).

**TABLE 3:** Results of section A: defined need and support for change.

	Section A: defined need and support for change	Mean score/5	SD
1	To what extent are innovation, experiential learning and quality student experiences clearly described as central to the mission and philosophy of your institution?	3.65	1.05
2	To what extent has your organisation clearly defined the need to consider SBE integration?	3.30	1.02
3	To what extent have administrators within your organisation communicated a clear strategic vision for SBE?	2.67	1.01
4	To what extent have administrators within your organisation provided a written commitment to SBE?	2.23	0.86
5	To what extent have administrators within your organisation provided funding to support the commitment to SBE?	2.35	0.94
6	To what extent does your organisation promote the need for SBE based on current evidence, standards and guidelines?	2.86	0.79
7	To what extent is SBE currently being used as a teaching modality in your organisation?	3.21	1.07
8	To what extent have the educators you work with articulated a need for SBE integration into the curriculum?	3.72	1.15
9	To what extent have the educators in your department verbalised a commitment to SBE integration into the curriculum?	3.37	1.06

Section B of the SCORS tool assessed the organisation’s readiness for culture change, and the results show the highest mean values of positive attitudes towards SBE (Question 10c, mean = 3.93) and to what extent do you believe that now is the right time to implement a culture change to support SBE (Question 18, mean = 4.26) (see Table 4).

**TABLE 4:** Results of section B: readiness for culture change.

	Section B: readiness for culture change	Mean score/5	SD
10	To what extent is there a critical mass of professionals who already possess strong SBE:		
a	Knowledge	2.81	0.90
b	Skills	2.72	0.87
c	Positive Attitudes	3.93	0.82
11	To what extent do administrators support culture change including the efforts required to implement and sustain SBE programme integration?	3.02	0.88
12	To what extent are there credentialed or trained simulationists who mentor/coach others, including, other simulationists?	2.16	0.94
13	To what extent does your department have individuals who model SBE best practice?	2.72	0.84
14	To what extent are staff/faculty proficient in the use of technology? (i.e., computer systems, AV and IT systems)	2.95	0.94
15	To what extent are there graduate level prepared researchers available to assist in research to develop new knowledge, appropriate to your department’s mission?	2.56	0.95
16	To what extent are librarians available within your institution to help search for evidence‐based practice and related simulation resources?	2.63	1.12
17	To what extent are your librarians accessed to search for evidence‐based practice and related simulation resources?	2.21	0.95
18	To what extent do you believe that now is the right time to implement a culture change to support SBE?	4.26	0.97

Section C of the SCORS tool focuses on time, personnel and resource readiness. The results show the availability of fiscal resources returned lower mean scores and that release time to lead integration of SBE reported the lowest mean value (Question 19c mean = 1.53) closely followed by fiscal resources for the development of spaces (Question 19d, mean = 1.98) (see Table 5).

**TABLE 5:** Results of section C: time, personnel and resource readiness.

	Section C: time, personnel and resource readiness	Mean score/5	SD
19	To what extent are fiscal resources available to support SBE in the following areas:		
a	Human resources (simulation personnel)?	2.05	0.71
b	Education?	2.02	0.79
c	Release time to lead integration of SBE?	1.53	0.76
d	Development of physical learning spaces?	1.98	0.70
e	Equipment?	2.23	0.74
20	To what extent do employees in your department have access to quality technology, including computers, audiovisual equipment and other institutional technologies?	3.21	1.05
21	To what extent is support available to learn and manage technologies that support education?	2.95	0.86
22	To what extent are there existing simulation champions (people who will go the extra mile to advance simulation) in the current environment among:		
a	Administrators	2.05	0.89
b	Clinicians	2.56	0.97
c	Educators	3.07	1.02
d	Technology Specialists	2.40	0.92
e	Administrative Assistants and Support Staff	1.74	0.87

Regarding decision making about SBE activities, it was identified that the most influential group was administrators (mean = 3.63) followed by educators (mean = 2.95) (see Table 6).

**TABLE 6:** Results of section D: sustainable education development to embed culture.

	Section D: sustainable education development to embed culture	Mean score/5	SD
23	To what extent is the measurement and sharing of outcomes part of the culture of the department in which you work?	2.79	0.82
24	To what extent are decisions regarding SBE influenced by:		
a	Clinicians	2.44	0.76
b	Educators	2.95	0.70
c	Administration	3.63	1.26

The overview of readiness per section results show that section C, time, personnel and resource readiness returned the lowest mean of 2.31, and section A, defined need and support for change, returned the highest mean of 3.04 (see Table 7).

**TABLE 7:** Overview of readiness.

	Overview of readiness per section	Mean score/5
A	Defined need and support for change	3.04
B	Readiness for culture change	2.96
C	Time, personnel and resource readiness	2.31
D	Sustainable practices to embed culture	2.95

The total overall score, which indicates the organisation’s readiness to implement an SBE programme, was 103.86, which indicates that the organisation is ‘somewhat’ ready to implement an SBE programme (see Table 8).

**TABLE 8:** SCORS summary and overall impression.

	SCORS summary impression	Mean score/5
25	Considering all the SCORS indicator scores, how would you rate your organisations’ readiness for SBE integration?	2.67

26	Looking back 6 months, how would you have rated your organisations’ readiness for SBE integration?	2.23
	Total overall score: Not ready: 0–36, a little: 37–72, somewhat: 73–108, moderately: 109–144, very much: 145–180	103.86

The classification of ‘somewhat ready’ reflects the SCORS framework, indicating that while there is recognition of the value of SBE and willingness to support its implementation, organisational readiness is not yet fully established and key structural elements remain underdeveloped highlighting the need for targeted organisational investment [21].

## 4. Discussion of Findings

The intention of this study was to assess staff perceptions of the organisation’s readiness to implement SBE programmes. What sets this initiative apart is its ambitious goal to integrate SBE across multiple health disciplines and geographically dispersed campuses. Importantly, the key themes that emerged were consistent across all groups, further justifying the decision to analyse the data collectively. As the study was primarily concerned with organisational readiness, this collective approach enabled readiness for SBE implementation to be examined at an institutional level while still acknowledging the complexity of coordinating simulation across diverse professional fields and remaining responsive to the distinct demographic and cultural contexts of each campus.

The research question was comprehensively addressed, and the findings are discussed as discrete themes aligned with the subsections of the SCORS tool. This unified perspective provides valuable insights into organisational readiness for SBE implementation while acknowledging the diversity of roles and settings represented within the participant cohort. Future research with larger, more balanced subgroup representation may offer further opportunities to explore potential variations across disciplines and campuses.

When considering a defined need and support for change, the results show that experiential learning and quality student experience are highly valued and central to the faculties mission and that SBE activities are currently used in practice and valued by participants. A defined need and support for change scored highly in areas of willingness to change and commitment to embrace SBE and quality outcomes for students. This represents an important enabler for SBE implementation. The high levels of willingness and commitment suggest that there is a strong desire within the organisation to implement and support the necessary changes, which is crucial for the advancement and effectiveness of the SBE programme.

A key component for any SBE programme to be successful is staff willingness to embrace change and be supported by the organisation. This organisational support can include policies, mission statements, learning objectives, and financial resources, which are aligned to the policies and procedures of the organisation [29]. Added to this, there is an acceptance of SBE activities across disciplines, which frequently utilise SBE as part of their teaching and curriculum integration. These findings align with broader nursing education literature, which highlights the importance of structured, evidence‐based teaching models, such as flipped classroom approaches (experiential learning), in enhancing student engagement, critical thinking and learning outcomes [6].

Readiness for culture change is a key driver for any change management initiative and is a critical factor in the success or failure of such initiatives [30]. The results of this study demonstrate that a critical mass of participants held positive attitudes towards change initiatives, suggesting that the timing is appropriate for implementing a culture change to support SBE. With this positive appetite for change, the organisation appears ready for this transformation. This supports Sharma et al.’s [29] finding that ‘openness to change’ is the precursor to readiness for change. Collectively, these findings suggest that the organisation has an important foundation from which to advance the implementation of SBE [29, 30].

However, there appears to be a lack of an organisational framework to support such activities, minimal strategic direction and communication from managers to provide vision, and poor clarity and commitment to SBE programmes. The results also show a lack of documented and financial commitment from the organisation to support SBE activities and a lack of organisational promotion of standards of best practice. Embedding a governance framework is crucial not only in the initial phases of SBE programmes but also for futureproofing, ensuring sustained growth over multiple years. Previous research has identified that this governance framework must align with the business case and strategic plan and address operational needs, orienting both current objectives and future aspirations [31–33]. These findings suggest that while formal decision‐making authority for SBE implementation was perceived to sit largely with administrators, educators also play an important role in influencing decisions through their curriculum knowledge, teaching expertise and understanding of learner needs. Faculty have identified the allocation of fiscal resources, release time to lead integration of SBE and development of physical learning spaces, as areas of concern demonstrated by consistently low score in these areas. These results would suggest that participants are dissatisfied with the financial allocation of funding and that more funding should be allocated to support SBE activities. These findings accord with studies that reported that a lack of financial support, limited coordination between departments, poor planning of physical spaces and inadequate annual budgeting of funds are contributing barriers to the successful implementation of simulation programme activities [4, 31, 34, 35]. Added to this, a lack of dedicated time for faculty and dedicated roles were also seen as major barriers in embedding quality simulation into higher education curricula, which also align with previous studies conducted [4, 31, 34, 35].

Participants agreed there was adequate access to technology and, to a lesser degree, support when learning or using the technology; however, there was an insufficiency when it came to the availability of financial support for human resources, release time to lead SBE integration and development of physical learning spaces. Interestingly, the results identified access to technology and hardware was not seen as a barrier to SBE readiness and that there was sufficient opportunity to use, learn and manage such technologies. This in conjunction with simulation champions within the education team supports the notion that the faculty are ready for change and can lead the change but require the resource to do so. This finding resonates with Hamstra’s [36] distinction between simulation centres as educational support systems and expressions of technology, suggesting that the organisation’s readiness challenge is less about access to equipment and more about the educational, organisational and workforce systems required to support effective SBE. This study extends Hamstra’s work by applying this distinction to a multidisciplinary, multi‐campus university context, showing that readiness for SBE implementation depends on the alignment of infrastructure with governance, faculty capability, curriculum integration, leadership support and a shared understanding of simulation as an educational strategy rather than a technology‐driven activity.

Additionally, educators were seen to be the champions of SBE activities, but administrators and administrative support staff did not rank as highly. In line with other studies, participants reported there were not enough trained staff in simulation across the faculty and that there is a lack of knowledge and skills to draw upon [5, 37, 38]. Development of knowledge and use of evidence‐based best practice were also considered to be lacking. Time, personnel and resource readiness fell below the standard expectation, which could be considered a barrier and an area for improvement and opportunity.

However, a lack of trained or credentialed staff presents a threat. While the desire to implement SBE is present, the ability to deliver is hampered by the lack of suitably trained staff. This is consistent with similar studies that have shown that a lack of trained staff had significant impact on both the quality of SBE programmes and staff confidence in delivering such programmes [7, 34, 38].

These findings align with several previous studies that demonstrate not allocating sufficient resources to time and personnel, jeopardises the success of SBE programmes [31, 34, 35, 37, 39].

Furthermore, the results showed that sustainable practices to embed culture also fell below the standard score, which indicates that this is an area for focus and improvement. The lack of sufficient sustainable practices may hinder the long‐term success of the programme, so it is crucial to focus efforts on improving this area. This has been viewed as a barrier in several other studies, which found that failure to embed sustainable practices hinders the success of SBE programmes [32, 39, 40].

### 4.1. Key Barriers to Organisational Readiness and Future Development

The results also revealed several barriers to the success of implementing SBE into the organisational practices. A lack of governance and formal commitment from administrators revealed a lack of readiness from the organisation and were barriers for the organisation, as was the perceived lack of fiscal commitment from the organisation. The inability to access suitably trained and credentialled staff was regarded as a barrier to the organisational readiness and an issue to be addressed. Arguably the most significant barrier is the inadequate fiscal allocation to support SBE implementation, its success and sustainability, in particular, the personnel required to support SBE, ongoing education into SBE activities, release time for faculty to lead integration of SBE and the appropriate development of learning spaces. These barriers while significant are areas for opportunity and intervention and should be used as a platform to address and be rectified, which will afford the opportunity to improve, enhance and grow SBE across the organisation. Regardless of the level of readiness, unless sufficient resources are devoted to SBE, and it will not be successful or sustainable.

### 4.2. Recommendations

In line with the best practice and current literature, it is recommended that the organisation is guided by the results provided and seek to develop a strategy, governance and operation model, which is aligned with the organisation’s principles [41, 42]. This strategy should include a clearly articulated rationale for SBE that defines its intended contribution to student learning, workforce preparedness, interprofessional collaboration, patient safety and educational quality [6, 11].

The operation model would identify priorities for SBE activities based on foundational principles, measure impact, integrate with existing systems and incorporate evidence‐based, student‐centred teaching approaches to enhance engagement and learning outcomes [6, 11]. This would include a governance and leadership model for simulation, which is embedded and supported across all levels of the organisation and is centralised to increase fiscal efficiencies and sustainability. Centralising resources would facilitate the establishment of a leadership team endowed with a clear vision and mission. This team would be mandated to oversee reporting functions, curriculum integration, spearhead faculty development initiatives and drive simulation programmes across the entire organisation. In this sense, the recommended model positions simulation not primarily as a technology‐based asset but as an organisationally driven, educational support system that enables curriculum integration, faculty development, evaluation and sustainable practice [36]. To demonstrate value, the model should incorporate outcome measures at multiple levels, including student engagement and learning outcomes, staff capability and confidence, simulation utilisation rates, curriculum integration, equity of access across campuses, patient safety and future patient care outcomes, cost‐effectiveness and alignment with accreditation or quality standards [21, 41, 42]. Establishing these measures would enable the organisation to evaluate the impact of SBE, demonstrate return on investment and communicate its broader value to the university, health services, students and the community [21, 41].

For nurse education leaders, nursing simulation programme managers and academic nursing managers, these findings underscore the importance of treating SBE as a strategic educational function rather than an isolated teaching activity. Nursing management has a key role in aligning simulation governance, faculty workload allocation, capability development, curriculum integration and evaluation processes to ensure SBE is sustainable within nursing programmes and contributes to broader multidisciplinary health education outcomes.

This approach positions simulation as a central function at the university and acknowledges the importance of developing a multi‐campus and multi‐disciplinary approach to the design and delivery of SBE.

### 4.3. Limitations

The study was conducted at a university based in Victoria, Australia. Therefore, staff at other universities might have differing responses. Although the study examined SBE across multiple health disciplines and campuses, the sample was limited to preregistration health programmes represented within the participating university. It did not include medicine, as this was not offered at the participating university, nor did it examine hospital‐based, postgraduate or continuing professional development simulation contexts, where simulation has distinct and well‐established communities of practice. Consequently, the findings may not fully reflect the organisational conditions, professional cultures, governance structures or simulation practices of all simulation environments. The response rate of 37% is also a limitation and may introduce non‐response bias, as the views of staff who did not participate may differ from those who completed the survey. Accordingly, the findings should be interpreted as reflecting the perceptions of respondents rather than as fully representative of all staff across the participating disciplines. While casual and sessional staff were excluded, the sample included permanent academic and professional staff with detailed organisational knowledge. Although the sample size and response rate were modest, all participating disciplines were represented, providing useful insights into organisational readiness for SBE within this multidisciplinary university context.

## 5. Conclusion

This research provides critical insights into the importance of identifying organisational readiness of a university to implement an SBE programme across multiple disciplines and multiple campuses. The study contributes to the literature by examining SBE readiness within a multidisciplinary and geographically dispersed university context while remaining responsive to local demographics and cultural contexts. Importantly, investigating the factors that enable or inhibit SBE implementation sheds light on the organisation’s appetite for investment in this initiative from both the business and educational needs.

The findings indicate that the organisation is ‘somewhat’ prepared to implement an SBE programme. Several enablers are present and active within the organisation, including a clearly defined need for change, support for the initiative, a willingness to embrace change and a commitment to achieving quality SBE outcomes for students. Nevertheless, the study also identified multiple barriers, such as a lack of time and personnel, insufficient resource allocation and challenges in practice.

Understanding these issues offers valuable insights into the organisation’s readiness. Based on these findings, the development of a clear strategy, aligned governance framework and practical centralised operational model will be essential to strengthen enablers, address existing barriers and support the successful delivery of a sustainable, high‐quality SBE programme. Realising this vision will require more than incremental adjustments; it demands a coordinated, institution‐wide approach supported by governance and systems that are both scalable and responsive to the unique needs of a multidisciplinary, multi‐campus context.

## Author Contributions

James Naismith: conceptualisation, methodology, investigation, formal analysis, writing–original draft and writing–review and editing.

Wendy Cross: supervision, conceptualisation, methodology and writing–review and editing.

Kerry Hood: supervision, conceptualisation, methodology and writing–review and editing.

Loretta Garvey: supervision, conceptualisation, methodology and writing–review and editing.

## Funding

This research received no specific grant from any funding agency in the public, commercial or not‐for‐profit sectors.

Open access publishing facilitated by Federation University Australia, as part of the Wiley ‐ Federation University Australia agreement via the Council of Australasian University Librarians.

## Conflicts of Interest

The authors declare no conflicts of interest.

## Data Availability

The data that support the findings of this study are available on request from the corresponding author. The data are not publicly available due to privacy or ethical restrictions.
